# 4D Flexible Atom-Pairs: An efficient probabilistic conformational space comparison for ligand-based virtual screening

**DOI:** 10.1186/1758-2946-3-23

**Published:** 2011-07-06

**Authors:** Andreas Jahn, Lars Rosenbaum, Georg Hinselmann, Andreas Zell

**Affiliations:** 1 University of Tübingen, Center for Bioinformatics Tübingen (ZBIT), Sand 1, 72076 Tübingen, Germany

## Abstract

**Background:**

The performance of 3D-based virtual screening similarity functions is affected by the applied conformations of compounds. Therefore, the results of 3D approaches are often less robust than 2D approaches. The application of 3D methods on multiple conformer data sets normally reduces this weakness, but entails a significant computational overhead. Therefore, we developed a special conformational space encoding by means of Gaussian mixture models and a similarity function that operates on these models. The application of a model-based encoding allows an efficient comparison of the conformational space of compounds.

**Results:**

Comparisons of our 4D flexible atom-pair approach with over 15 state-of-the-art 2D- and 3D-based virtual screening similarity functions on the 40 data sets of the Directory of Useful Decoys show a robust performance of our approach. Even 3D-based approaches that operate on multiple conformers yield inferior results. The 4D flexible atom-pair method achieves an averaged AUC value of 0.78 on the filtered Directory of Useful Decoys data sets. The best 2D- and 3D-based approaches of this study yield an AUC value of 0.74 and 0.72, respectively. As a result, the 4D flexible atom-pair approach achieves an average rank of 1.25 with respect to 15 other state-of-the-art similarity functions and four different evaluation metrics.

**Conclusions:**

Our 4D method yields a robust performance on 40 pharmaceutically relevant targets. The conformational space encoding enables an efficient comparison of the conformational space. Therefore, the weakness of the 3D-based approaches on single conformations is circumvented. With over 100,000 similarity calculations on a single desktop CPU, the utilization of the 4D flexible atom-pair in real-world applications is feasible.

## Background

Sorting and comparing molecules from chemical databases represent two of the key tasks in cheminformatics [1]. The sorting of such databases, with respect to a given set of queries (molecules) and similarity functions, is known as virtual screening (VS). The goal of VS is to enrich molecules with similar properties (e.g., biological activity) to the query molecules and to discover new chemical entities in a small fraction of the database. To ensure the desired properties (e.g., biological activity) and to evaluate the success of the VS run, it is necessary to further analyze the enriched molecules by means of biological assays. The success of a VS run consists of two different aspects. First, the enriched molecules should have similar properties as the query molecules. Second, the discovery of new chemical entities that consist of different scaffolds in comparison with the query molecules, and, therefore represent an information gain. Based on the focus on a relevant subset of the database and the possible structural information gain, VS experiments represent a fundamental approach in the drug discovery pipeline [2,3].

In the last two decades a plethora of different similarity functions were proposed [4,5], and the development of new functions is still an open field of research. All similarity functions can be categorized by the dimension of the applied representation of molecules. 1D similarity functions are based on molecular property counts such as molecular weight or number of hydrogen bond acceptors. 2D approaches make use of the adjacency matrix of the molecular graph, and, therefore they are also called topological-based approaches. MOLPRINT2D [6], substructure-based fingerprints like BCI [7] and DAYLIGHT [8] as well as the MACCS [9] keys are well known 2D similarity methods. Those topological or structural fingerprints yield promising results with respect to the enrichment of active molecules, but often lack the ability to discover new chemical entities [10]. 3D similarity functions are based on the shape [11-14] or geometrical distance information [15-17] of molecules. Information of the conformational ensembles of molecules extends the 3D-based methods and can be seen as 4D approaches [18,19].

Based on the key-lock principle of Hermann Emil Fischer, it could be expected that the shape of molecules plays an important role for the biological activity. However, the shape of a molecule is not unique, but rather a function of internal parameters like the torsion angles. Hence, each rotatable bond represents a degree of freedom and increases the number of possible shapes (conformations) of the molecule. The resulting space, which contains all possible conformations, represents the conformational space of the molecule. Based on this increased complexity, it is not surprising that several literature studies reported a more robust VS performance of 2D methods in comparison to 3D approaches [20,21]. Further arguments for 2D methods are their simplicity and speed [22].

In a comprehensive study, Venkatraman et al. [21] investigated the performance of different 2D and 3D methods on a wide range of pharmaceutically relevant targets. The results of the study underpin the predominant opinion that 2D-based approaches are superior to 3D approaches with respect to the enrichment of active molecules. The performance of the 2D and 3D approaches with respect to the knowledge gain by means of the discovery of new chemical entities was not evaluated by the study. A possible reason for the inferior performance of 3D methods is the geometric information that is based on one conformation of the molecule [21]. One opportunity to improve the performance of 3D methods is to apply the 3D methods on different conformations of the molecules and use the mean or maximum similarity value. The drawback of this workaround is the quadratic increase in computation time, which scales with the number of conformations. To address this runtime issue, it is necessary to perform the similarity calculation on the complete conformational ensemble in one step in a feasible manner. These limitations of 3D approaches also affect the performance of instance-based machine learning QSAR/QSPR models. To improve the robustness of those QSAR/QSPR models, we developed a 4D-based approach that is able to compare the conformational space of molecules within one step in feasible time [23]. The results showed that our approach produces robust models that are superior to similar 3D and 2D approaches. Given the fact that the reasons for the inferior performance of 3D-based methods seem to be similar in both applications (VS and QSAR/QSPR), it is possible that our 4D-based approach is also able to increase the VS performance in comparison to 2D and 3D methods.

The aim of this study is to evaluate our 4D approach as VS similarity function on a variety of literature VS benchmark data sets. Additionally, we compare the results to state-of-the-art 2D and 3D approaches to assess the performance of our method. We employed VS performance metrics that measure the chemotype enrichment performance to reduce the influence of artificial enrichment. The results show a robust performance of our approach in comparison to state-of-the-art 2D and 3D approaches. Therefore, our conformational space comparison is able to reduce the weakness of 3D-based methods without the time-demanding pair-wise comparison of individual conformations.

## Methods

This section describes our 4D flexible atom-pair (4D FAP) similarity measure on the conformational space of molecules. To allow an efficient comparison of the conformational space of molecules, our approach needs a special encoding of the conformational ensembles, which can be seen as a preprocessing step. First, We describe our conformational space encoding. Afterwards, a modified Expectation Maximization (EM) algorithm will be presented that computes generative models, which represent the behavior of the molecules in their conformational space. Finally, the actual similarity calculation, which operates on the preprocessed molecules, will be explained.

### Conformational Space Encoding

To ensure a fast comparison of the conformational space of molecules, it is necessary to transform the complex information of the conformational space of molecules into a representation that is suitable for the integration into fast similarity functions. Therefore, we decompose the information of the conformational space into small portions. Given a conformational sampling *C_M _*of molecule *M *with |*M*| heavy atoms, the encoding is based on the distance behavior of atom-pairs in the conformational space. Hence, the conformational space *C_M _*of molecule *M *is segmented into the distance behavior of  atom-pairs.

**Figure 1:**
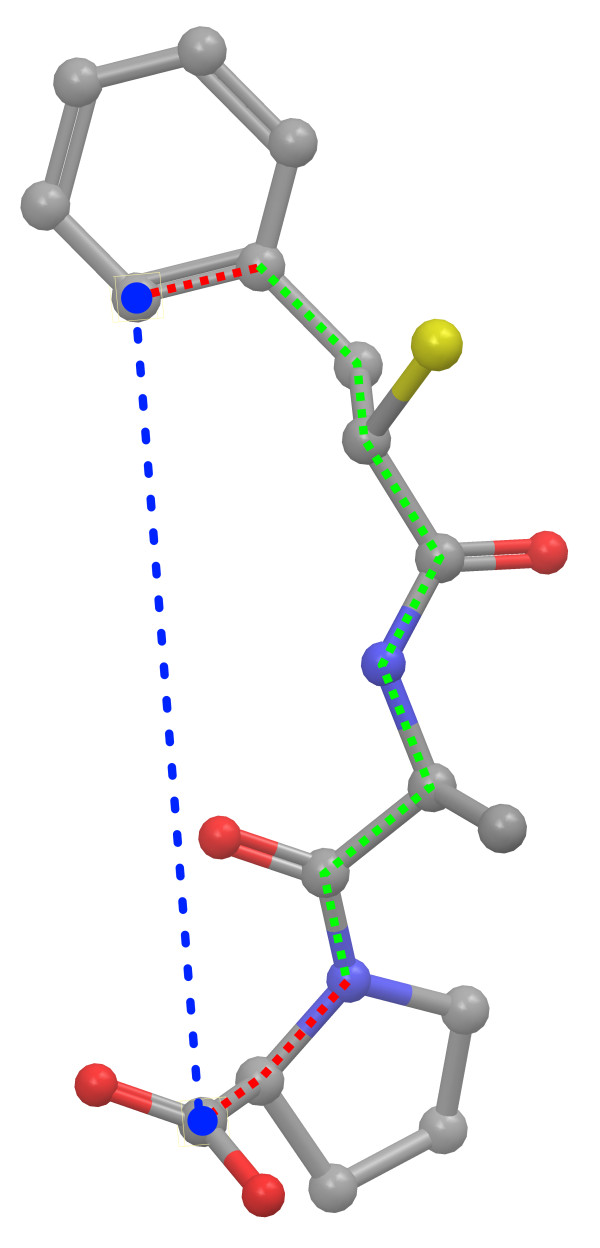
**Shortest flexible atom-pair path**. Exemplary visualization of an atom-pair of the marked atoms. The shortest topological path is depicted by the red and green dotted lines. A red line represents a rigid bond, whereas a green line marks a rotatable bond. The last bond of the path (bond from the heterocycle to the carbon of the carboxyl group) is treated as a rigid bond because a rotation of this bond has no influence on the geometric distance of the atom-pair.

Figure 1 represents exemplarily an atom-pair and the corresponding geometric distance. Not all of the  atom-pairs of a molecule *M *have a flexible distance behavior in the conformational space. The distance relation of neighboring atoms or atoms of a ring system only shows a small variability of the distance. Therefore, our encoding separates the atom-pairs into two disjoint classes: The flexible and the rigid atom-pair class. The separation is realized by a heuristic that employs the number of rotatable bonds in the shortest path of the corresponding molecular graph. Figure 1 visualizes the shortest path of the marked atom-pair. A bond is supposed to be rotatable if it is a single bond and not a bond of a ring system. If the number of rotatable bonds in the shortest path is ≥ 1, the atom-pair represents a flexible, otherwise a rigid atom-pair. Terminal rotatable bonds (rotatable bonds that are adjacent to one of the atoms that form the atom-pair) are not counted in the heuristic because a rotation of such a bond has no influence on the distance relation of the atoms (Figure 1).

Given the class of flexible atom-pairs from the heuristic above, our encoding measures the distance of each atom-pair and conformation of the given conformational sampling *C_M_*. This results into atom-pairs that have |*C_M_*| distance values, where |*C_M_*| represents the number of sampled conformers of molecule *M*. We refer to the atom-pairs containing the distance information in the conformational space as distance profiles.

**Figure 2:**
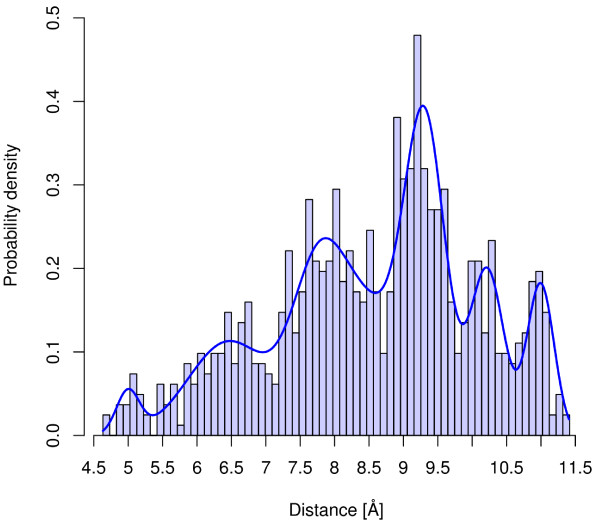
**Atom-pair distance distribution**. Histogram-based visualization of the distance distribution of the marked atom-pair of Figure 1. The line represents the corresponding GMM that models the distance behavior of the atom-pair in the conformational space.

These distance profiles can be visualized by means of normalized histograms, which represent the relative frequency of observing the corresponding atom-pair distance within a binned distance range. A histogram-based visualization of the distance profile from the atom-pair of Figure 1 can be seen in Figure 2. The application of histograms in a similarity function entails two major drawbacks. First, the binning size represents a parameter and has substantial impact on the resulting similarity value. Second, the storage of the information needs more space than a model-based encoding. Therefore, we decided to describe the distance behavior in the conformational space by means of Gaussian Mixture Models (GMMs). After the encoding of the distance profiles as GMMs the preprocessing of the molecules is finished.

### Gaussian Mixture Models and Parameter Estimation on Distance Profiles

Mixture models are probabilistic models that represent a complex distribution based on a linear combination of individual sub-distributions. Applying Gaussian distributions as sub-distributions in a mixture model yields a GMM as given in Equation 1, where *p*(**x**) represents the probability density at the point **x**, *π_c _*determines the weight of the c-th Gaussian distribution, and  depicts the c-th Gaussian distribution with mean ***μ****_c _*and covariance matrix **Σ***_c_*.(1)

GMMs are generative models for real-world data and involve two advantages in our application. First, a conformational ensemble of a molecule represents only a discrete sampled approximation of the complete conformational space. Therefore, the flexible atom-pairs contain a series of sampled distance values. A generative model, fitted to the distance values, represents a continuous function, and, therefore describes a more realistic model in comparison to discrete or binned values. Second, the models can be efficiently stored because only the model parameters are necessary for a similarity calculation between such models. A drawback of the GMMs is the parameter estimation for a given data set. Given the distance values of a flexible atom-pair, it is necessary to fit the parameters *π_c_*, ***μ****_c_*, **Σ***_c_*, and *C *(number of Gaussian components) of Equation 1 to the distance values. A popular approach to determine the parameters of a mixture model is the Expectation Maximization (EM) algorithm [24]. This algorithm is based on the maximum likelihood framework and optimizes the objective function given in Equation 2.(2)

The EM algorithm represents an iterative process that consists of two steps. The first step (E-step) evaluates the responsibilities that the k-th component of the GMM was responsible for generating the n-th (**x***_n_*) data point of the given data set **X **(Equation 3).(3)

The second step (M-step) updates the parameters of the GMM on the basis of the responsibilities of the previous E-step (Equation 4-7).(4)(5)(6)(7)

These two steps are repeated until a predefined convergence criterion is reached. The EM algorithm optimizes the parameters of the GMM and guarantees a local optimum solution. Therefore, it is necessary to execute the EM algorithm with different initial parameters to avoid a model from a local optimum with an inferior likelihood value.

The EM algorithm estimates the parameters of a GMM based on a predefined number of Gaussian components. A suitable number of components is crucial for a useful model. Therefore, a model selection step that determines an optimal number of components is necessary. To reduce the risk of overfitting (high number of Gaussian components), several model selection criterions, such as the Bayesian information criterion [25] or the Akaike information criterion [26], were proposed that penalize an increased number of components. This model selection step involves a significant runtime overhead and can be avoided if the number of sub-distributions can be estimated. In our application, a GMM has to model the distance behavior of the corresponding atom-pair in the conformational space. The distance behavior of an atom-pair can be seen as a function of the flexibility of the shortest path in the molecular graph.

Therefore, the number of flexible bonds in the shortest path (as applied to classify the atom-pairs) can also be applied as a heuristic to determine the number of Gaussian components for the GMM. In an earlier study we already presented the comparable performance of the heuristic in comparison to model selection criterions [23]. This heuristic avoids the model selection step and reduces the runtime of the preprocessing step. Figure 2 presents the GMM that models the distance behavior of the atom-pair in Figure 1. The presented EM algorithm assumes that all samples of the data set are equally important for the final model. Transferred to our application this means that each conformation has the same influence on the final model. Based on a thermodynamic point of view, this assumption of equal influence holds if all conformations of the ensemble have the same energy. To emphasize the influence of low energy conformations on the final model, we developed an extension of the EM algorithm that integrates the importance of each sample into the optimization process. In an earlier study, our modified EM algorithm generated improved QSAR/QSPR models in comparison to models based on equally weighted GMMs [27].

### Boltzmann Weighted Expectation Maximization Algorithm

To increase the importance of low energy conformations on the final GMMs of a molecule, we apply the normalized Boltzmann distribution as given in Equation 8 to determine a probability value for a given conformer. Δ**E***_n _*represents the energy offset of the n-th conformer to the global optimum of the conformational ensemble, R presents the gas constant, and T the temperature of the canonical ensemble.(8)

These probability values have to be integrated into the EM algorithm and modify the objective function as outlined in Equation 9, where **E **symbolizes a vector containing the energy values of the conformers and *p*(**E***_n_*) depicts the probability of the n-th conformation.(9)

The E-step (computation of the responsibilities) remains unchanged, and, therefore the responsibilities are calculated as stated in the Equation 3. However, the equations of the M-step (update of the parameters) need the integration of the probability values as listed in the Equations 10-13.(10)(11)(12)(13)

**Figure 3:**
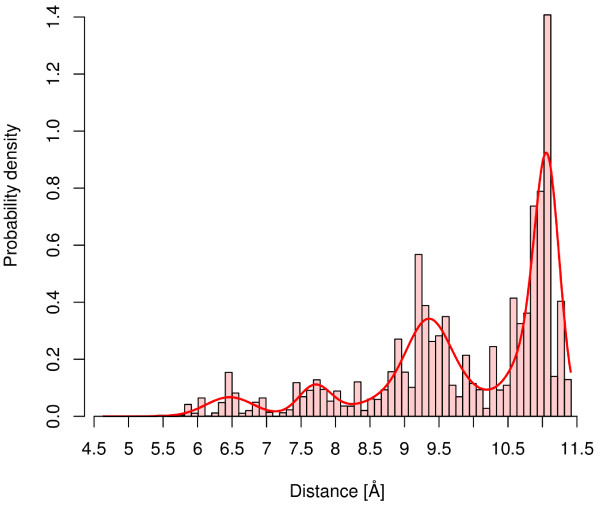
**Boltzmann weighted atom-pair distance distribution**. Boltzmann weighted histogram-based visualization of the distance distribution of the atom-pair of Figure 1. The line describes the probability density of the GMM that was computed by the Boltzmann weighted EM algorithm.

Based on the described modifications, the EM algorithm computes GMMs that represent the distance behavior of atom-pairs as a function of the frequency of observing an atom-pair at a certain distance as well as the probability that the canonical ensemble will occupy these states (conformations). Figure 3 visualizes a weighted (probabilities of the conformations) histogram-based representation of the same atom-pair visualized in Figure 2. The Boltzmann weighted model of Figure 3 shows that the distances at 9.25 Å and 11 Å are energetically favorable, and, therefore the probability density is increased in comparison to the unweighted model of Figure 2. In contrast, the conformations with low range distances have higher energy values and, as a consequence, the corresponding probability densities are reduced.

### 4D Flexible Atom-Pair Similarity Function

After the preprocessing of the molecules (encoding the distance distributions by means of GMMs) the actual 4D similarity calculation can be conducted. The similarity function operates on the molecular graph (adjacency matrix) and the GMMs of the flexible atom-pairs. Therefore, the conformational ensemble of the molecules is not further needed.

**Figure 4:**
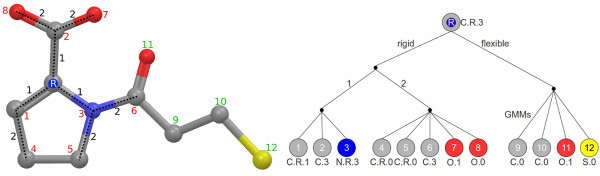
**Flexible atom-pair tree**. The left molecule represents the example molecule for the tree on the right side. The white 'R' marks the atom that serves as root atom (point of origin for the atom-pairs) for the tree. The black numbers symbolize the topological distance for the rigid atom-pairs. The red and green numbers correspond with the leaf numbers of the tree on the right side. The red or green color of these atom numbers indicates the membership of the atom to the rigid or flexible sub-tree, respectively.

In a first step, the 4D FAP creates for each heavy atom of the molecule an atom-pair prefix tree. This data structure represents an efficient approach for search and comparison operations and was already applied as a data structure for an atom-pair-based similarity measure [28,29]. Each prefix tree has one atom as root node and contains all atom-pair information to the remaining |*M*| - 1 heavy atoms of the molecule (leaves of the tree). The preprocessing step divides the atom-pairs into two disjoint classes. Therefore, an atom-pair tree *T *contains the two different sub-trees *R *and *F *for the rigid and flexible atom-pair class, respectively. The rigid sub-tree *R *contains the information of the rigid atom-pairs that were not modeled by GMMs. To increase the information content of the rigid atom-pairs, the sub-tree additionally contains the topological distance information of each atom-pair. An example of such an atom-pair tree can be seen in Figure 4.

The nodes in these prefix trees can be labeled by any arbitrary atom typing scheme. We applied a labeling function that consists of three different elements. The first element is the element symbol of the atom. A ring flag indicates the membership to a ring system. The final value is the result of the number of neighboring heavy atoms minus the number of neighboring hydrogen atoms.

Given the prefix trees of two molecules *A *and *B*, the 4D FAP computes a similarity matrix *S*, where each entry represents the similarity value between two atom-pair trees. Based on the two different sub-trees, an entry *S^ij ^*in the similarity matrix is the sum of two distinct similarity calculations  on the sub-trees. Hence, the 4D FAP utilizes different similarity functions for the sub-trees.

The rigid sub-tree contains the labels of the atoms and a topological distance value. This type of information represents nominal features and enables the use of simple similarity functions. We applied the Tanimoto similarity function as stated in Equation 14. *R_i _*and *R_j _*represent the rigid atom-pair sub-trees of the *i*-th and *j*-th atom, respectively.(14)

The comparison of the flexible atom-pair sub-tree consists of two different similarity functions. Given the flexible atom-pair sub-trees *F_i _*and *F_j _*containing |*F_i_*| and |*F_j_*| flexible atom-pairs (number of leaves in the sub-tree). The first function compares the atom labels of two given flexible atom-pairs AP*_n _*∈ *F_i _*and AP*_m _*∈ *F_j _*as presented in Equation 15. *l_m _*and *l_n _*represent the atom labels of the atom-pairs. The function is a Dirac function on the atom labels and returns a value of 1.0 if the labels are equal and 0 otherwise.(15)

The second similarity function compares the behavior of the atom-pairs in the conformational space. For this purpose, a correlation measure on GMMs is applied as denoted in Equation 16, where *g_m _*and *g_n _*symbolize the GMMs of the flexible atom-pair AP*_m _*and AP*_n_*, respectively.(16)

The assembly of both similarity functions for flexible atom-pairs results in Equation 17 and represents the similarity function for the flexible atom-pair sub-tree.(17)

Unlike the similarity function for the rigid atom-pair sub-tree, the similarity function for the flexible atom-pair sub-tree is not based on nominal features. Therefore, Equation 17 performs a pair-wise comparison of all atom-pairs and sums up the individual similarity scores. To avoid overestimated similarity values of sub-trees with an increased number of flexible atom-pairs, the similarity value is normalized by Equation 18(18)

After computation of the similarity matrix *S*, which contains all pair-wise similarity values of the atom-pair trees, the 4D FAP computes a final similarity value based on the matrix *S*. The original 4D FAP, as applied in QSAR/QSPR studies [23,27], sums up the entries of the *S *matrix and normalizes the sum to obtain a value in the range [0.0, 1.0]. Another possibility to compute a final similarity value represents the optimal assignment. This approach was introduced into the field of cheminformatics by Fröhlich et al. [30,31] and applied as a VS similarity function in a previous study [28].

Preliminary experiments (not published) showed that an optimal assignment on the matrix *S *improves the VS performance in comparison to the normalized summation of the matrix elements. Therefore, we changed the final computation step of the 4D FAP to perform an optimal assignment on the matrix *S *as stated in Equation 19. Given the molecules *A *and *B *(with |*A*| < |*B*| w.l.o.g.), *π *represents a function that maps each value of *i *∈ [1, ..., |*A*|] on a value in the range [1, ..., |*B*|] in such a way that the sum of the similarity entries is maximized. The final sum of the optimal assigned similarity values of the atom-pair trees is also normalized by Equation 18. We refer to the optimal assignment-based variant of the 4D FAP as 4D FAP_OA_.(19)

## Experimental

In this section we initially characterize the applied VS benchmark data sets as well as their preparation step. Afterwards, the protocol for the conformational sampling of the molecules as well as a short description of the VS evaluation metrics follow. Finally, we present a brief overview of literature VS methods that were applied to classify the results of the 4D FAP_OA_.

### Data sets

To evaluate the 4D FAP_OA _on a wide range of pharmaceutically relevant targets, we employed the directory of useful decoys (DUDs) release 2 [32]. These data sets were introduced as a benchmark data set compilation for the evaluation of docking algorithms [33]. Ligand-based VS, based on similarity values to a query structure, can be afflicted with an analogue enrichment bias. This bias results from the enrichment of structurally similar molecules with respect to the query structure. These similar structures represent only a limited information gain, and, therefore the results of the experiment will have an analogue enrichment bias.

To reduce this bias and to enable a fair comparison between similarity-based and docking-based algorithms, Good and Oprea applied a lead-like filter [34] on the data sets and clustered the actives [35]. These filtered and clustered data sets were already applied in a ligand-based VS study [28] and are publicly available [36]. Table 1 shows a complete overview of the 40 targets and the number of actives and decoys for the DUD release 2 and the filtered variant. For the VS experiments we applied the target ligands as query structure for the respective active and decoy data set. The data sets were not further modified to allow a fair comparison of the results.

**Table 1:** DUD data sets

	Filtered sets		Original sets	
		
target	actives	decoy	actives	decoys
ACE	46	1796	49	1797
AChE	99	3859	107	3891
ADA	23	927	39	927
ALR2	26	986	26	995
AmpC	21	786	21	786
AR	68	2848	79	2854
CDK2	47	2070	72	2074
COMT	11	468	11	468
COX-1	23	910	25	911
COX-2	212	12606	426	13289
DHFR	190	8350	410	8366
EGFr	365	10303	475	15996
ER_agonist_	63	2568	67	2570
ER_antagonist_	18	1058	39	1448
FGFr1	71	3462	120	4550
FXa	64	1633	146	5743
GART	8	155	40	879
GPB	52	2135	52	2947
GR	32	2585	78	2947
HIVPR	4	9	62	2038
HIVRT	34	1494	43	1519
HMGR	25	1423	35	3478
HSP90	23	975	37	979
InhA	57	2707	86	3266
MR	13	636	15	634
NA	49	1713	49	1874
P38	137	6779	454	9140
PARP	31	1350	35	1351
PDE5	26	1697	88	1977
PDGFrb	124	5603	170	5980
PNP	25	1036	49	1036
PPAR_γ_	6	40	85	3117
PR	22	920	27	1041
RXR*_α_*	18	575	20	750
SAHH	33	1346	33	1346
SRC	98	5679	159	6319
thrombin	23	1148	72	2456
TK	22	891	22	891
trypsin	9	718	49	1664
VEGFr2	48	2712	88	2906

Number of active and decoy compounds for the original and filtered DUD targets.

The evaluation of similarity functions by means of the DUD data sets represents a retrospective evaluation. Analogous to the "Kubinyi paradox" [37] of QSAR models, the solely retrospective evaluation possibly implies the risk that the development of new methods or the improvement of existing approaches will increase their retrospective performance at the expense of the prospective performance. However, the DUD data sets contain over 100,000 molecules for 40 different targets. Consequently, the evaluation on all 40 data sets is based on an increased molecular diversity in comparison with the usually smaller and less diverse benchmark data sets of QSAR experiments. Therefore, the risk of an inferior prospective performance of VS similarity functions as a result of their optimization for the retrospective performance is reduced but still present.

### Conformational sampling

To create the conformational ensembles of the molecules, we applied the ConfGen tool of Schrödinger [38]. Recent studies showed the ability of ConfGen to compute reasonable conformers of molecules [39,40]. The tool provides four standard parameter schemes that sample the conformational space at different resolutions. To compute useful GMMs in the preprocessing step, it is necessary to sample the conformational space at a high resolution. Therefore, we modified the 'comprehensive' parameter scheme of ConfGen to further increase the resolution. We reduced the heavy atom rmsd for distinct conformers from 0.5 Å to 0.1 Å. This modification results in more conformers but does not increase the runtime of the conformational sampling. The energy values, which are necessary for the Boltzmann weighted GMMs, were computed by the OPLS 2005 force-field with standard parameters.

The applied conformational sampling algorithm as well as the force-field model have a major impact on the final results of the 4D FAP_OA_. Different conformational sampling algorithms compute different sets of conformers, which in turn yield different atom-pair distance profiles. The force-field computes an energy value for each conformer and determines the weight of each measured atom-pair distance. As a result, a different conformational sampling protocol will yield different GMMs of the atom-pairs. Hence, the computed similarity values differ and will probably change the results. However, the aim of this study is not the evaluation of the impact of different conformational sampling protocols on the 4D FAP_OA_, but the evaluation of the 4D FAP_OA _as a VS similarity function based on the given protocol.

### Evaluation metrics

To evaluate the performance of our 4D FAP_OA _approach, we applied different standard evaluation metrics. The receiver operating characteristic (ROC) curve represents a function that plots the true positive rate as a function of the false positive rate. The area under the ROC curve (AUC) represents a quantification of the curve and facilitates an easier comparison of results. The AUC is calculated as given in Equation 20, where *N*_actives _depicts the number of actives, *N*_decoys _represents the number of decoys, and  describes the number of decoys that are higher ranked than the *i*-th active structure. The received value is in the range [0.0, 1.0], where 0.5 indicates a random performance.(20)

The AUC metric represents a measure that evaluates the performance on the complete data. However, a major goal of VS experiments is the enrichment of active structures in a small fraction of the database. Therefore, it is necessary to apply additional metrics that focus on the early enrichment behavior. A common metric for the early enrichment problem is the enrichment factor at a predefined fraction of the data set (*x*%) as given in Equation 21.(21)

The enrichment factor depends on the number of actives, and, therefore it is not a robust metric. Korff et al. [41] proposed the relative enrichment factor (REF) as stated in Equation 22 to remove the dependency on the number of actives structures.(22)

The enrichment of active structures that are based on different scaffolds emerged to an additional important goal of VS experiments. All metrics that evaluate the so-called chemotype enrichment are based on a clustering of the active structures into different chemotypes (scaffolds). Mackey and Melville showed the integration of the scaffold information into common VS metrics [42]. We decided to apply the arithmetic weighting on the ROC enrichment as given in Equation 23. Based on the information of the clustering, a structure *i *obtains a weight that is inversely proportional to the number of structures (*N_j_*) in the cluster .  represents a binary function that returns 1 if the *i*-th active structure of the *j*-th cluster is contained in the first *x*% of the data set.(23)

The evaluation metrics listed above represent only a small fraction of possible metrics. Other popular metrics for the early enrichment evaluation are the BEDROC [43] score or the enrichment factor. To enable future comparisons with the presented results of the 4D FAP_OA_, we computed for each target of the filtered DUD data set a result file that contains several additional VS metrics (e.g., BEDROC score at nine predefined alpha values). Additionally, the files contain the complete ranking of the molecules that allows the computation of the VS metric of choice. The 40 result files are contained in the additional file 1 of this study.

### Literature Similarity Functions

We employed a wide range of different 2D and 3D similarity approaches to assess the performance of the 4D FAP_OA_. Due to the fact that we compare our approach to 20 other approaches, we only mention the name of the method and the applied type of information. For a comprehensive description we refer to the original publications.

Different optimal assignment approaches were already evaluated on the filtered DUD data sets in an earlier publication [28]. The best approach of this study was a two-step hierarchical assignment (2SHA) that first operates on a substructure level and afterwards on the atomic level. A second approach of that study optimally assigns the atom-pair (OAAP) environment trees and represents a similar 3D concept in comparison to the 4D FAP_OA_. The optimal assignment kernel (OAK) [30,31] and its flexibility extension, the OAK_FLEX _[44], were also evaluated in this earlier publication.

Cheeseright et al. [45] introduced FieldScreen as a multiconformer-based VS tool. FieldScreen utilizes a database that contains conformers of each molecule. Therefore, it operates on a conformational ensemble in a similar way as the 4D FAP_OA _and represents an interesting reference approach. FieldScreen employs four different types of locally optimized molecular field points to compute a similarity value between two given molecules.

Venkatraman et al. conducted a comparison study in which a plethora of different 2D and 3D approaches were evaluated on the original as well as the filtered DUD data sets [21]. We compared the performance of the 4D FAP_OA _to the main results of this study. The study conducted by Venkatraman et al. employed the 2D fingerprint methods: OPENBABEL [46], DAYLIGHT [8], BCI [7], MACCS [9], and MOLPRINT2D [6]. As 3D-based approaches they utilized ROCS [12] with two different scoring schemes ROCSS (shape only) and ROCS_SC _(shape and chemistry). The EON [47] approach compares the electrostatic fields computed by the Poisson-Boltzmann equation and was also evaluated using two different parameterizations. EON_SE _is based on the shape and the electrostatic, whereas EON_SCE _additionally uses chemical information.

SHAEP [14] is based on a maximum common subgraph approach that is employed to perform a superposition of the molecules. The method operates only on the shape of molecules (SHAPE_S_) or on the shape and the electrostatic (SHAPE_SE_). The Ultrafast Shape Recognition (USR) [17] employs four distance relations of each atom and computes the first three moments of each distribution to obtain 12 descriptor values for each molecule. ESHAPE3D is based on a heavy atom distance matrix that is employed to compute fingerprints. The ESHAPE_HYD _alternatively uses the hydrophobic heavy atoms.

PARAFIT [13] computes a similarity value based on spherical harmonic expansions of molecular surfaces. Another important class of similarity functions are the pharmacophore-based approaches. These approaches operate on an abstract representation of the molecules by means of pharmacophore features. These features are divided into different classes (e.g., hydrogen bond acceptor, aromatic, or hydrophobic) and represent important interaction points of molecules. The distance relation between these pharmacophore features plays an important role and can be measured in a topological or geometrical manner. Therefore, the pharmacophore-based approaches can also be divided into 2D- and 3D-based approaches. Korff et al. [41] compared different structure- and ligand-based VS approaches on the DUD data sets. This study contains two different pharmacophore-based methods. The topological pharmacophore point histogram (TopPPHist) computes for each pair of pharmacophore classes a distance histogram based on the topological distances. Therefore, the TopPPHist represents a 2D-based pharmacophore approach. Finally, the distance histograms are converted into a descriptor vector. The Flexophore approach [1,41] computes geometrical and binned distance histograms for each pharmacophore point pair based on a representative set of given conformers. The final comparison between two molecules is similar to the maximum common subgraph-isomorphism because the pharmacophore points together with the distance histograms form complete graphs.

## Results and Discussion

The results section is divided into four different subsections. The first subsection compares the results of the 4D FAP_OA _approach with other optimal assignment-based approaches that were already evaluated on the DUD data sets [28]. The second part is based on the results of Venkatraman et al. [21] and compares the average performance of the 4D FAP_OA _with 15 state-of-the-art 2D and 3D approaches. Afterwards, a comparison with the pharmacophore-based approaches of Korff et al. [41] follows. The final subsection focuses on the performance difference between 3D approaches on multiple conformers and our 4D FAP_OA _approach.

### Comparison with other Optimal Assignment Methods

The comparison with other optimal assignment methods measures the influence of the applied information type on the final performance. The OAAP represents the comparable 3D approach in comparison with the 4D FAP_OA_, and, therefore directly measures the performance gain of the 4D extension. As an early enrichment metric we applied the awROCE_5%_, which also assesses the chemotype enrichment performance. To reduce the bias introduced by a low number of chemotypes, we only applied data sets that have at least 15 different chemotypes. The AUC value was applied to evaluate the performance on the complete data sets.

Table 2 shows the results of the four optimal assignment methods and the 4D FAP_OA_. The direct comparison of the OAAP and the 4D FAP_OA _indicates that the 4D FAP_OA _outperforms the OAAP on 10 out of 13 data sets with respect to both performance measures. The OAAP is superior to the 4D FAP_OA _on the COX2, HIVRT, and the PDGFrb data sets. These three data sets are more rigid data sets with respect to the number of rotatable bonds of the query compounds. The query compounds of COX2, HIVRT, and PDGFrb have 5, 9, and 7 rotatable bonds, respectively. In comparison, the most flexible data sets are the ACE and EGFr data sets with 18 and 14 rotatable bonds, respectively. The correlation between the performance gain on the AUC metric  and the number of rotatable bonds of the query structure amounts to 0.54. Based on the equation , where *n *is the number of samples (13 data sets) and *r *the correlation, the probability that both variables (AUC performance gain and flexibility of query compounds) result in such a correlation if there is no true correlation of the variables (*ρ *= 0.0) is 0.0277. Therefore, the correlation is significant (*p *= 0.05) and indicates that the performance gain of the 4D FAP_OA _is a function of the flexibility of the data set. In comparison to the other optimal assignment methods there is no correlation apparent. However, the OAK, OAK_FLEX_, and 2SHA are based on a different type of information (local atom similarity based on atom and bond features), and therefore, a direct comparison of the correlations is not meaningful.

**Table 2:** Optimal assignment methods results

	OAK		OAK_FLEX_		2SHA		OAAP		4D FAP_OA_	
					
target	awROCE_5%_	AUC	awROCE_5%_	AUC	awROCE_5%_	AUC	awROCE_5%_	AUC	awROCE_5%_	AUC
ACE	12.1	0.78	12.1	0.76	11.6	0.82	8.0	0.58	**12.2**	**0.88**
AChE	3.9	0.69	4.4	0.71	5.4	0.74	4.0	0.71	**7.6**	**0.75**
CDK2	2.6	0.57	2.6	0.47	**3.5**	0.50	**3.5**	0.55	**3.5**	**0.77**
COX2	9.0	0.88	8.8	0.89	9.7	0.87	**12.2**	**0.93**	11.9	0.89
EGFr	11.6	0.76	11.3	0.75	12.1	0.74	7.3	0.51	**18.0**	**0.99**
FXa	2.1	0.43	1.1	0.51	2.6	0.59	2.1	0.58	**3.2**	**0.64**
HIVRT	3.3	0.53	3.3	0.48	3.5	0.60	**5.1**	**0.65**	2.3	0.58
InhA	8.6	0.54	5.7	0.53	**9.4**	0.63	7.0	0.57	7.8	**0.66**
P38	4.3	0.43	4.0	0.44	**5.0**	**0.75**	2.9	0.45	3.1	0.68
PDE5	2.3	0.46	1.4	0.41	2.7	0.47	1.4	0.38	**3.6**	**0.69**
PDGFrb	4.9	0.44	4.9	0.38	4.5	0.34	**8.6**	0.42	4.9	**0.66**
SRC	3.7	0.67	4.5	0.64	**6.4**	**0.72**	1.0	0.45	2.7	0.51
VEGFr2	1.3	0.28	1.3	0.30	**4.5**	0.47	2.6	0.39	3.2	**0.67**

avg. rank	3.35	3.54	3.81	3.77	**2.15**	2.62	3.38	3.35	2.31	**1.58**

awROCE5% and AUC performance of the OAK, OAKFLEX, 2SHA, OAAP, and 4D FAPOA. The approaches were evaluated on the 13 filtered DUD data sets that entail at least 15 different chemotypes in the active data set.

Bold values indicate the best results on the corresponding data set and metric. The last row contains the average rank of the corresponding approach with respect to the metric and the other approaches.

The comparisons of the 4D FAP_OA _with all other optimal assignment approaches show that the 4D FAP_OA _outperforms all other methods on 6 and 9 data sets with respect to the awROCE_5% _and AUC, respectively. These results yield a best average rank of 1.58 for the 4D FAP_OA _with respect to the AUC. For the awROCE_5% _results the 2SHA achieves the best average rank of 2.15 followed by the 4D FAP_OA _with an average rank of 2.31. In a direct comparison the 4D FAP_OA _outperforms the 2SHA approach on 7 data sets, whereas the reverse case only occurs on 5 data sets. To conclude, the 4D FAP_OA _shows a robust performance on 13 data sets. Considering the results of the complete data sets (AUC) the 4D FAP_OA _outperforms all other optimal assignment methods. The ability of 4D FAP_OA _to early enrich different scaffolds is comparable with the 2SHA approach.

The encoding of the conformational space should be most beneficial if the flexibility of the query structure is high. Therefore, we discuss the results on the two data sets with the most flexible query compounds, the ACE and EGFr data set, in more detail.

**Figure 5:**
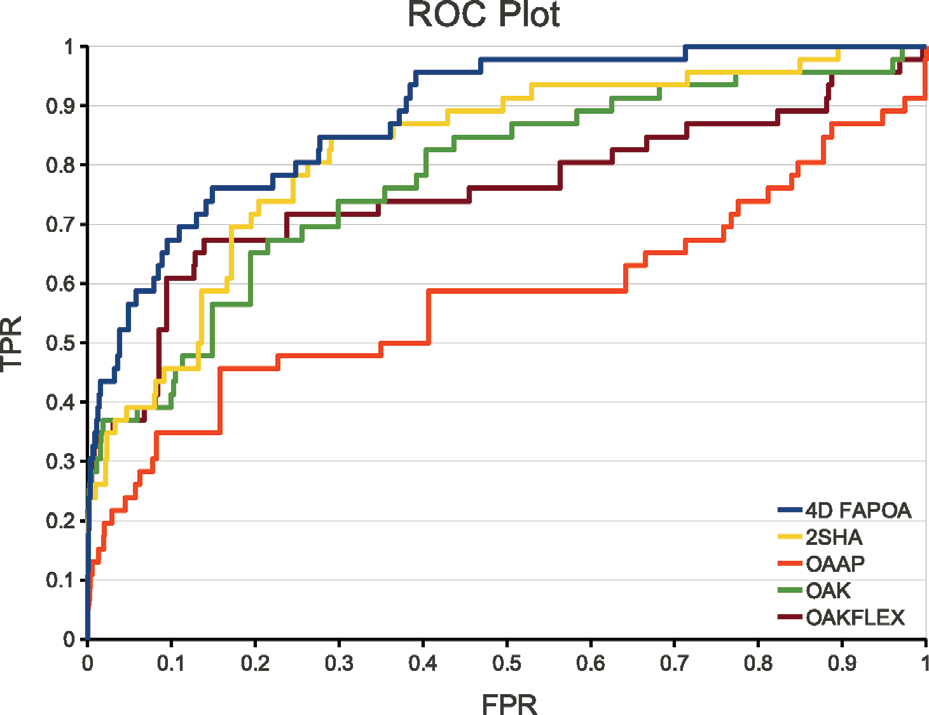
**ROC plot on ACE**. ROC plot of all optimal assignment methods on the filtered ACE data set. TPR and FPR denote the true positive rate and false positive rate, respectively.

Figure 5 shows the ROC plot of all optimal assignment methods on the ACE data set. The curve of the 4D FAP_OA _passes always above the other curves with the exception of the 2SHA curve between 0.3 and 0.4 false positive rate. In the early enrichment range (0.0 - 0.1 false positive rate) the 4D FAP_OA _shows a strong increase of the true positive rate without any longer phases of stagnation (horizontal elements in the curve). The other optimal assignment methods show a similar behavior till ≈ 0.03 false positive rate, but they stagnate until ≈ 0.1 false positive rate. Therefore, the 4D FAP_OA _has an offset of the true positive rate of nearly 0.2 in comparison to the other methods. The 4D FAP_OA _is also the first approach that is able to retrieve all actives of the data set (≈ 0.7 false positive rate). The second approach that retrieves all actives is the 2SHA approach at a false positive rate of ≈ 0.9. The comparable 3D approach (OAAP) is always inferior in comparison to the 4D FAP_OA_.

To evaluate the chemotype discovery on the complete data set, we plotted the fraction of the discovered chemotypes as a function of the fraction of the ranked data set. A chemotype is considered as discovered if one compound of the chemotype is ranked.

**Figure 6:**
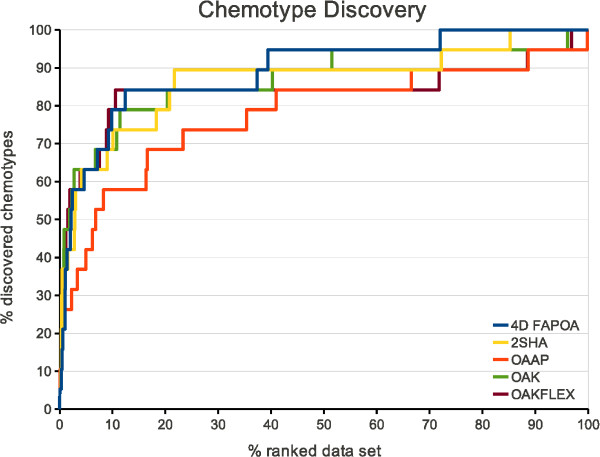
**Chemotype discovery on ACE**. Chemotype discovery of all optimal assignment methods on the filtered ACE data set.

Figure 6 presents the chemotype discovery of all optimal assignment approaches on the ACE data set. The curves of the 4D FAP_OA_, 2SHA, OAK, and OAK_FLEX _show a similar behavior over the complete data set. Only the OAAP has an inferior chemotype discovery rate until ≈ 40% of the data set. Therefore, the information gain by discovering new chemotypes is increased by the 4D FAP_OA _in comparison to its similar 3D method (OAAP). However, the other approaches that are based on a different type of information (OAK, OAK_FLEX_, and 2SHA) show a similar discovery rate.

**Figure 7:**
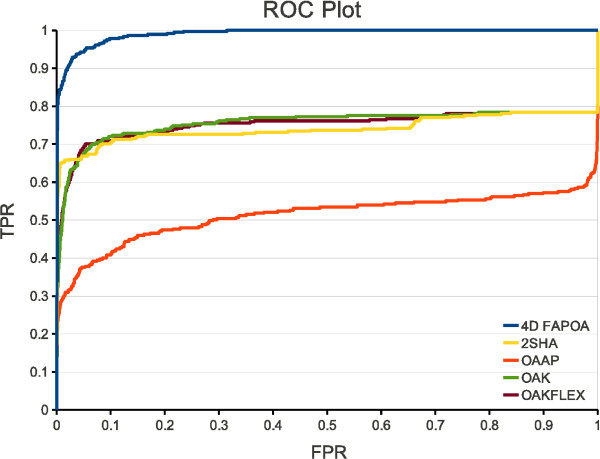
**ROC plot on EGFr**. ROC plot of all optimal assignment methods on the filtered EGFr data set.

**Figure 8:**
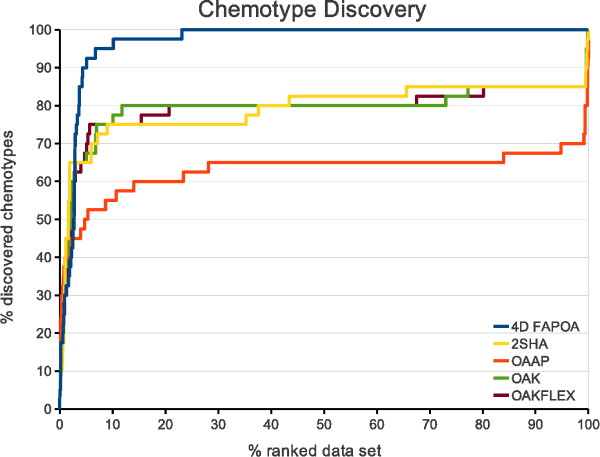
**Chemotype discovery on EGFr**. Chemotype discovery of all optimal assignment methods on the filtered EGFr data set.

The ROC plots and the chemotype discovery on the EGFr data set can be seen in the Figures 7 and 8, respectively. In both figures a considerably performance gain of the 4D FAP_OA _is apparent. The 4D FAP_OA _is able to retrieve all actives within ≈ 30% of the data set (Figure 7). All chemotypes were discovered within 23% of the data set (Figure 8). All other optimal assignment methods retrieve at least 20% of the actives and at least 15% of the chemotypes within the last percent of the data set. In comparison to the OAAP the performance gain of the 4D FAP_OA _is approximately twice that of the OAK, OAK_FLEX_, and 2SHA. Therefore, our encoding of the conformational space entails a significant performance gain on the EGFr data set.

Another important property of a VS similarity function is the computation performance. To enable a VS experiment on a real-world database, the VS similarity function should be able to process a reasonable number of compounds in a feasible time. All presented VS similarity functions that are based on the optimal assignment approach were developed at our department, and, therefore we are able to perform a fair comparison of the computation time. We computed the average computation time of each optimal assignment method on the 13 data sets, which were used in Table 2, to approximate a reasonable performance for drug-like compounds.

The 4D FAP_OA _approach has an averaged performance of 38.8 ± 27.56 similarity calculations per second. This computation time is based on preprocessed molecules (GMMs already computed). The OAK yields 27.34 ± 3.40 calculations per second, whereas its flexibility extension (OAK_FLEX_) computes 41.03 ± 7.32 molecules per second. The OAAP represents the fastest approach with 51.49 ± 18.07 computations per second. In contrast, the 2SHA is the slowest method with a throughput of 14.04 ± 1.78 per second. All calculations were done on a Core2Duo CPU with 2 GHz using one core and 1 GB memory. As a result, the 4D FAP_OA _is fast enough to screen over 100,000 molecules within one hour on a desktop CPU using only one core. The similarity calculation can be easily parallelized to further increase the throughput, and, therefore the approach should be fast enough for real-world applications.

The preprocessing step (conformational sampling and GMM calculation) represents an additional computational task of our approach. However, the preprocessing step has only to be computed once for each molecule. Additionally, the computation of different conformers (conformational sampling) is often necessary for different tasks in the drug discovery pipeline. Furthermore, our encoding is a model-based encoding that reduces the memory usage in a database in comparison to the storage of multiple conformers of a molecule.

### Comparison with State-of-the-Art 2D and 3D Approaches

In this subsection we compare the performance of the 4D FAP_OA _with different state-of-the-art 2D and 3D approaches. Venkatraman et al. [21] conducted a comprehensive evaluation of 15 different literature methods on the DUD data sets. The study contains the averaged (over all 40 data sets) relative enrichment factors at 1%, 5%, and 10% as well as the AUC values for each method. Unfortunately, the study lacks any evaluation metric that rates the chemotype discovery of the approaches. Therefore, the results in this section are only based on the early enrichment (relative enrichment factors) and the performance on the complete data set (AUC). All results are based on the filtered data sets and compiled in Table 3.

**Table 3:** Average REF and AUC performance

method	REF_1%_	REF_5%_	REF_10%_	AUC	avg. rank
BABEL	44.4 ± 28.4	41.1 ± 25.4	49.6 ± 26.6	0.74	3.25
DAYLIGHT	43.9 ± 28.7	41.8 ± 25.8	52.2 ± 26.7	0.74	2.75
MACCS	30.5 ± 25.7	29.7 ± 22.8	39.6 ± 23.3	0.69	7.0
BCI	**46.7 **± **31.7**	41.3 ± 28.5	49.1 ± 29.7	0.74	2.75
MOLPRINT2D	34.5 ± 28.3	33.8 ± 26.9	40.9 ± 30.2	0.70	6.0

PARAFIT_S_	19.1 ± 20.3	24.4 ± 20.1	33.0 ± 22.4	0.67	12.5
ROCS_SC_	36.8 ± 29.7	35.2 ± 27.1	44.0 ± 28.7	0.72	5.0
ROCS_S_	27.3 ± 25.7	27.8 ± 22.4	35.2 ± 24.1	0.65	10.25
EON_SCE_	24.2 ± 26.5	24.8 ± 24.1	33.3 ± 24.1	0.68	10.375
EON_SE_	22.9 ± 25.4	24.7 ± 21.5	32.2 ± 22.8	0.68	11.625
SHAEP_SE_	29.0 ± 25.5	27.2 ± 22.1	35.3 ± 23.7	0.67	9.75
SHAEP_S_	28.1 ± 26.6	27.2 ± 22.1	35.5 ± 23.8	0.67	8.875
USR	12.7 ± 15.6	16.2 ± 13.9	24.3 ± 17.6	0.61	15.0
ESHAPE3D_HYD_	24.0 ± 27.6	23.1 ± 20.8	27.8 ± 23.4	0.54	13.75
ESHAPE3D	14.1 ± 16.8	13.0 ± 9.8	18.6 ± 12.7	0.42	15.75

4D FAP_OA_	46.0 ± 33.1	**45.4 **± **30.1**	**53.5 **± **31.0**	**0.78**	**1.25**

Averaged relative enrichment factors and AUC values of different 2D and 3D approaches as well as the 4D FAP_OA _method. Bold values indicate the best result with respect to the corresponding metric. The right column lists the average rank of each method with respect to the other approaches in the table. The horizontal lines separates the 2D-, 3D-, and 4D-based approaches.

The results of Table 3 confirm that the 2D approaches are more robust in comparison to the 3D methods. Only the ROCS_SC _is able to yield comparable results in comparison to the MACCS keys and MOLPRINT2D. The 4D FAP_OA _is able to utilize the GMMs as a source of reasonable information, and, therefore the approach yields the best results with respect to the relative enrichment factor at 5% and 10% as well as the AUC metric. Only the BCI approach is able to marginally improve the results with respect to the relative enrichment factor at 1%. The best performance of the 4D FAP_OA _on three out of four metrics results in the best average rank of 1.25. The BCI and DAYLIGHT fingerprints yield an average rank of 2.75 and represent the best 2D-based approach. ROCS_SC _is the best 3D-based approach with an average rank of 5.0, and, therefore higher ranked as the 2D-based approaches MOLPRINT2D (6.0) and the MACCS keys (7.0). All other 3D-based methods are inferior in comparison to the 2D-based approaches. To conclude, the 4D FAP_OA _benefits from the conformational space information and is able to yield the best average performance of all methods.

### Comparison with Pharmacophore-Based Approaches

Korff et al. [41] evaluated the TopPPHist and the Flexophore approach on the 40 targets of the DUD data sets. The early enrichment performance was assessed by the relative enrichment factor at 1% of the data set. To evaluate the chemotype enrichment, Korff et al. counted the discovered chemotypes within the enriched data set fraction with respect to the chemotype definition of Good and Oprea [35]. Table 4 lists the relative enrichment factors and the number of discovered chemotypes for each of the 40 data sets of the DUD.

**Table 4:** Relative enrichment factors and chemotype discovery of pharmacophore-based approaches and the 4D FAP_OA_.

	TopPPHist		Flexophore		4D FAP_OA_	
target	REF_1%_	Chem_1%_	REF_1%_	Chem_1%_	REF_1%_	Chem_1%_
ACE	65.01	**8**	**75.84**	**8**	**75.84**	5
AChe	**57.54**	**4**	55.04	3	57.53	**4**
ADA	0.0	0	0.0	0	**41.41**	**1**
ALR2	9.79	1	9.79	1	**19.59**	1
AmpC	74.35	1	86.74	1	**99.13**	1
AR	44.4	**2**	27.32	1	**68.19**	1
CDK2	23.52	**4**	23.52	3	**41.94**	**4**
COMT	20.88	1	20.88	1	**62.63**	1
COX-1	21.39	1	**64.17**	**4**	64.1	**4**
COX-2	69.66	9	96.79	7	**99.16**	**13**
DHFR	**96.86**	4	91.03	**9**	91.16	6
EGFr	70.56	2	73.6	12	**97.14**	**13**
ER_agonist_	45.66	2	41.86	**5**	**72.05**	3
ER_antagonist_	**47.07**	**2**	26.9	**2**	**47.07**	1
FGFr1	0.0	0	**12.92**	0	10.71	**4**
FXa	5.1	1	**23.78**	**4**	8.49	2
GART	**10.99**	0	**10.99**	0	10.88	0
GPB	72.99	3	36.5	3	**96.7**	3
GR	26.47	1	26.47	**3**	**36.36**	**3**
HIVPR	**4.78**	0	4.78	0	0.0	0
HIVRT	**38.49**	2	**38.49**	**3**	38.41	2
HMGR	66.05	1	99.08	**2**	**99.14**	**2**
HSP90	**99.6**	2	69.72	2	98.43	2
InhA	86.52	5	**95.47**	**6**	**95.47**	**6**
MR	0.0	0	0.0	0	**77.04**	**1**
NA	31.2	1	20.8	1	**57.2**	1
P38	0.0	0	**22.27**	**1**	20.85	**1**
PARP	7.22	1	**14.43**	**2**	0.0	0
PDE5	58.45	2	58.45	1	**96.85**	**3**
PNP	28.14	2	**93.81**	**4**	55.3	3
PPAR*_γ_*	87.28	0	**96.63**	0	87.45	0
PR	0.0	0	9.45	**1**	**37.45**	**1**
RXR*_α_*	12.99	1	**77.92**	1	51.95	1
SAHH	58.01	1	36.26	1	**79.77**	1
SRC	1.96	1	0.0	0	**7.72**	**2**
TK	32.86	1	**54.76**	**2**	43.81	1
trypsin	**5.95**	0	**5.95**	0	5.84	**1**

mean	37.34	1.78	43.31	2.54	**55.48**	**2.65**
avg. rank	2.35	2.30	2.04	1.93	**1.61**	**1.80**

Relative enrichment factors and number of discovered chemotypes of two different pharmacophore-based approaches and the 4D FAP_OA_. The last two rows contain the mean value and the average rank of the corresponding approach. Bold values indicate the best result with respect to the target and metric.

With respect to the early enrichment performance the TopPPHist and the Flexophore approach achieved an average relative enrichment factor of 37.34 ± 31.38 and 43.31 ± 33.25, respectively. The application of the 4D FAP_OA _resulted in an average relative enrichment factor of 55.45 ± 33.26 and increased the performance of the Flexophore approach by over 20%. However, based on their abstract representation of molecules, one of the strengths of pharmacophore-based approaches is the ability to discover new chemical entities. This abstraction from the query scaffold can be seen in the chemotype discovery results of Table 4. The Flexophore approach needs ≈ 20% less active compounds to discover a similar amount of chemotypes (94) in comparison with the 4D FAPOA (98). The 2D-based TopPPHist discovered only 66 chemotypes over all 40 data sets and showed an inferior chemotype discovery in comparison with the 4D-based approaches (Flexophore, 4D FAP_OA_).

### Comparison with Multiple Conformer Approaches

The results of the previous sections demonstrated the inferior performance of 3D-based approaches in comparison with 2D-based methods. A common technique to tackle this deficit of 3D approaches is to utilize multiple conformers and average or use the maximum of all pair-wise similarity values. The number of necessary similarity computations scales with *O*(*n*^2^), where *n *represents the number of conformers of the molecules. Therefore, this technique implies a significant increase in computation time. However, the averaging over multiple conformers increases the available information content of the 3D-based approaches to a level that is similar in comparison to the 4D FAP_OA_. The 4D FAP_OA _has a model-based description of the conformational space, whereas the 3D-based approaches explicitly have the conformational space. Consequently, a comparison of the 4D FAP_OA _with 3D-based approaches on multiple conformers represents an interesting comparison based on a equal source of information.

Venkatraman et al. [21] evaluated the ROCS_SC _(best 3D-based approach of Table 3) in three additional experiments on the unfiltered DUD data sets with different ensembles of size 10, 100, and 1000 conformers per molecule. Table 5 lists in detail the AUC performance of the ROCS_SC _on different ensemble sizes and the AUC results of the 4D FAP_OA_. The table also contains the AUC results of the ROCS_SC _on one given conformation as a baseline to evaluate the performance gain of the multiple conformer setup.

**Table 5:** Average AUC of ROCS_SC _with multiple conformers and the 4D FAP_OA_

		ROCS_SC_			4D FAP_OA _AUC
		
target	AUC_1_	AUC_10_	AUC_100_	AUC_1000_	
ACE	0.69	0.85	0.82	0.77	**0.89**
AChE	0.76	0.75	0.77	**0.78**	0.72
ADA	0.63	**0.76**	0.60	0.59	0.67
ALR2	0.45	0.47	0.49	0.50	**0.63**
AmpC	0.77	0.86	0.88	0.88	**0.90**
AR	0.81	0.80	0.79	0.79	**0.89**
CDK2	**0.78**	0.70	0.69	0.67	0.75
COMT	0.32	0.27	0.33	0.34	**0.97**
COX-1	0.62	0.62	0.58	0.57	**0.63**
COX-2	**0.95**	0.94	**0.95**	**0.95**	0.93
DHFR	0.68	0.45	0.91	0.89	**0.99**
EGFr	0.81	0.82	0.95	0.95	**0.98**
ER_agonist_	0.92	0.93	**0.94**	**0.94**	0.85
ER_antagonist_	0.94	0.97	**0.98**	**0.98**	0.92
FGFr1	0.53	0.61	0.51	0.45	**0.63**
FXa	0.61	0.49	**0.66**	0.64	0.62
GART	0.43	0.50	0.77	**0.84**	0.82
GPB	0.84	0.93	0.94	0.94	**0.97**
GR	0.81	0.81	0.77	0.76	**0.92**
HIVPR	**0.71**	0.61	0.58	0.61	0.21
HIVRT	0.71	0.71	**0.72**	0.71	0.56
HMGR	0.76	0.90	0.94	0.93	**0.97**
HSP90	0.69	0.71	0.66	0.64	**0.86**
InhA	0.72	**0.81**	0.78	0.79	0.69
MR	0.83	0.86	0.86	0.85	**0.91**
NA	**0.97**	0.96	**0.97**	**0.97**	**0.97**
P38	0.46	0.49	0.48	0.48	**0.75**
PARP	0.63	0.59	0.58	0.58	**0.77**
PDE5	0.68	0.58	0.59	0.56	**0.78**
PDGFrb	0.41	0.39	0.30	0.28	**0.60**
PNP	0.56	0.58	0.88	0.89	**0.94**
PPAR_γ_	0.87	0.68	0.74	0.91	**0.96**
PR	0.74	0.73	0.68	0.69	**0.94**
RXR*_α_*	0.88	**0.98**	0.97	0.95	**0.98**
SAHH	0.96	0.96	**0.98**	**0.98**	**0.98**
SRC	0.51	0.50	0.39	0.34	**0.53**
thrombin	0.54	**0.66**	**0.66**	0.55	0.59
TK	0.68	0.84	0.88	**0.89**	0.87
trypsin	0.41	0.49	0.57	0.65	**0.80**
VEGFr2	0.61	0.54	0.44	0.39	**0.64**

avg. rank	3.55	3.26	2.96	3.2	**2**

AUC values of ROCS_SC _(AUC(1)), ROCS_SC _on multiple conformer data sets (AUC(10), AUC(100), and AUC(1000)), and the 4D FAP_OA _on the original DUD data sets. The number in brackets indicates the number of sampled conformers. Bold values indicate the best results with respect to the data set. The last row contains the average rank of each approach on all 40 data sets with respect to the other approaches. ROCS_SC _results were taken from Venkatraman et al. [21]

The average AUC of the ROCS_SC _increases from 0.692 (AUC(1)) over 0.703 (AUC(10)) to 0.725(AUC(100)). The results on 1000 conformers are marginally inferior (average AUC(1000) of 0.722) in comparison to the results on 100 conformers. As a result, the ROCS_SC _slightly benefits from the additional information content of multiple conformers. However, the average AUC of the 4D FAP_OA _is 0.80, and, therefore superior in comparison to all four ROCS_SC _setups. These results are verified by the average ranks of the approaches. The 4D FAP_OA _is able to achieve the best AUC value on 27 out of 40 data sets and demonstrates its robust performance on a wide range of pharmaceutically relevant targets. The best ROCS_SC _setup (100 conformers) yields on eight data sets the best result. Please note that the different average AUC values in Table 3 and 5 are the result of the applied data sets (filtered DUD in Table 3 and unfiltered in Table 5).

**Figure 9:**
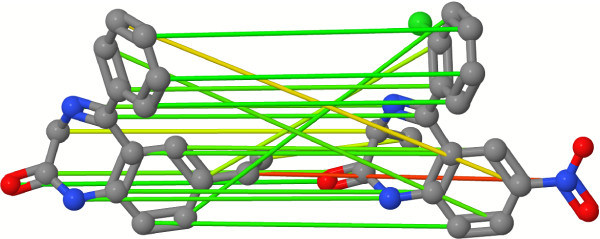
**Optimal assignment with topological errors**. Example mapping with several topological errors. Figure was taken from Jahn et al. [28]

Despite the robust and superior performance of the 4D FAP_OA _on the majority of the 40 data sets, the weak performance on the HIVPR data set is conspicuous. The HIVPR data set has an average number of heavy atoms of 36.3 and represents the data set with the largest compounds of all 40 DUD data sets. The 4D FAP_OA _entails an optimal assignment step to compute a final similarity value based on the atom-pair tree similarity matrix *S*. If the approach computes the similarity value between the query compound and a data set compound, the *i*-th row of *S *represents the atom-pair tree with the *i*-th atom of the query compound as root node. Analogously, this applies to the *j*-th column of *S *and the *j*-th atom of the data set compound. The optimal assignment step maps each atom of the query compound onto an atom of the data set compound. With an increased size of atoms the number of possibilities (possible mappings) scales with *O*(*n*!), where *n *is the number of heavy atoms. This increase also increments the risk of a topological error in the assignment step. Topological errors are assignments that do not preserve a substructure mapping (e.g., atoms of a ring are assigned to atoms of different rings). Figure 9 shows a mapping with several topological errors. These topological errors maximize the final similarity value, but from a chemical point of view these mappings are questionable. Therefore, these errors can negatively influence the ranking of the compounds on the HIVPR data set.

The FieldScreen approach by Cheeseright et al. [45] represents a VS similarity function that applies four different types of locally optimized field points and operates on a multiconformer database. Therefore, it also operates on a comparable information content as our 4D FAP_OA _approach. Cheeseright et al. evaluated the FieldScreen approach on the filtered DUD data sets and applied the chemotype information on the result metrics. The results of the FieldScreen approach as well as the 4D FAP_OA _are listed in Table 6.

**Table 6:** awROCE and AUC results of FieldScreen and the 4D FAP_OA_

	FieldScreen		4D FAP_OA_	
		
data set	awROCE_5%_	AUC	awROCE_5%_	AUC
ACE	4.7	0.67	**12.2**	**0.88**
AChE	7.3	**0.76**	**7.6**	0.75
CDK2	0.8	0.47	**3.5**	**0.77**
COX2	10.4	**0.92**	**11.9**	0.89
EGFr	9.5	0.84	**18.0**	**0.99**
FXa	**5.4**	**0.74**	3.2	0.64
HIVRT	**5.1**	**0.70**	2.3	0.58
InhA	6.5	**0.71**	**7.8**	0.66
P38	0.5	0.33	**3.1**	**0.68**
PDE5	**4.8**	0.66	3.6	**0.69**
PDGFrb	3.8	0.29	**4.9**	**0.66**
SRC	2.5	0.45	**2.7**	**0.51**
VEGFr2	**3.5**	0.48	3.2	**0.67**

mean	4.98	0.62	**6.5**	**0.72**
avg. rank	1.69	1.62	**1.31**	**1.38**

The approaches were evaluated on awROCE_5% _and AUC results of FieldScreen [45] and the 4D FAP_OA_.

The approaches were evaluated on the 13 filtered DUD data sets that entail at least 15 different chemotypes in the active data set. Bold values indicate the best results with respect to the corresponding metric and data set. The last two lines contain the average performance and average rank of each approach.

The 4D FAP_OA _yields a superior early enrichment performance (awROCE_5%_) on 9 out of 13 data sets. Concerning the performance on the complete data set (AUC) our approach outperforms FieldScreen on 8 data sets. The 4D FAP_OA _is able to increase the mean early enrichment and complete data set performance by ≈ 30% and ≈ 16%, respectively. The major improvements of FieldScreen in comparison to the 4D FAP_OA _are on the FXa and HIVRT data sets. These data sets also consist of larger molecules, and, therefore the risk of topological errors is increased and is probably a reason for the inferior 4D FAP_OA _performance.

To conclude, the best 3D-based approach of Table 3 (ROCS_SC_) could increase the performance if it is applied on multiple conformer data sets. However, the performance gain was not strong enough to reach the results of the 4D FAP_OA_. The comparison with the FieldScreen approach yields similar results and underpinned the robust performance of the 4D FAP_OA_. The detailed evaluation of the results reveals a weakness of our approach if the compounds of a data set have an increased number of heavy atoms. This weakness is likely the result of the optimal assignment step and was already reported as a weak point of optimal assignment approaches [28]. Nevertheless, the 4D FAP_OA _represents a robust similarity measure for small and medium sized drug-like compounds.

## Conclusions

We presented a VS similarity function that operates on GMM encoded conformational space information. Our approach is able to compare the conformational space of molecules within one step, and, therefore avoids the application of time-consuming averaging techniques. The approach was already applied in QSAR experiments and demonstrated its robust performance in comparison to similar 3D-based QSAR models [23,28].

The aim of this study was to evaluate our approach as VS similarity function. Therefore, we compared the results of the 4D FAP_OA _with 20 other 2D- and 3D-based approaches. Additionally, we applied two approaches (ROCS_SC _and FieldScreen) that operate on multiple conformers to provide a comparison of approaches that are based on a similar information content.

The results showed that our approach is able to achieve superior results on a wide range of pharmaceutically relevant targets. Even the best 3D approach, with respect to the results of Venkatraman et al. [21], applied on multiple conformers is inferior in comparison to our approach.

The preprocessing, which is necessary to encode the conformational space information by means of GMMs, represents an additional computational step. However, all compounds have only be computed once and the encoded models need less space in comparison to the storage of conformational ensembles. The computational speed of the actual similarity function is fast enough to screen over 100,000 compounds within one hour on a standard desktop CPU with one core. Therefore, our approach should meet the requirements of real-world VS applications.

The complete source code of the preprocessing tool (computing GMMs based on conformational ensembles) as well as the 4D FAP_OA _similarity function are publicly available on our department website http://www.cogsys.cs.uni-tuebingen.de/software/4DFAP.

## List of abbreviations

ACE: angiotensin-converting enzyme; AChE: acetylcholinesterase; ADA: adenosine deaminase; ALR2: aldose reductase; AmpC: AmpC *β*-lactamase; AR: androgen receptor; CDK2: cyclin-dependent kinase 2; COMT: catechol O-methyltransferase; COX-1: cyclooxygenase-1; COX-2: cyclooxygenase-2; DHFR: dihydrofolate reductase; EGFr: epidermal growth factor receptor; ER: estrogen receptor; FGFr1: fibroplast growth factor receptor kinase; FXa: factor Xa; GART: glycinamide ribonucleotide transformylase; GPB: glycogen phosphorylase *β*; GR: glucocorticoid receptor; HIVPR: HIV protease; HIVRT: HIV reverse transcriptase; HMGR: hydroxymethylglutaryl-CoA reductase; HSP90: human heat shock protein 90; InhA: enoyl ACP reductase; MR: mineralo-corticoid receptor; NA: neuraminidase; P38: P38 mitogen activated protein; PARP: poly(ADP-ribose) polymerase; PDE5: phosphodiesterase 5; PDGFrb: platelet derived growth factor receptor kinase; PNP: purine nucleoside phosphorylase; PPAR*_γ_*: peroxisome proliferator activated receptor *γ*; PR: progesterone receptor; RXR*_α _*retinoic X receptor *α*; SAHH: S-adenosyl-homocysteine hydrolase; SRC: tyrosine kinase SRC; TK: thymidine kinase; VEGFr2: vascular endothelial growth factor receptor.

## Competing interests

The authors declare that they have no competing interests.

## Authors' contributions

AJ designed and developed the main part of the 4D FAP_OA_, has written the manuscript, participated in the design of the experiments and the discussion of the results. LR participated in the design of the experiments and the discussion of the results. GH contributed to the development of the 4D FAP_OA_, participated in the design of the experiments and the discussion. AZ participated in the design of the 4DFAP_OA_, the design of the experiments, and the discussion of the results.

## Supplementary Material

Additional file 1 Archive of the 4D FAP_OA _result files. This is a Gzip compressed Tar archive containing the result files of the 4D FAP_OA _on the filtered DUD data sets. The result files are tab-separated plain text files including the following information: method name, active data set with size, cluster information and the distribution of the active molecules over the clusters, decoy data set with size, ratio active:decoy, AUC, awAUC, BEDROC scores for predefined alpha values as suggested by Truchon and Bayly [43], enrichment factors, relative enrichment factor [41], ROC enrichments, awROC enrichments at predefined false positive fractions, chemotype enrichment, ROC and awROC data points, and the ranking of each structure to compute other VS metrics. Click here for file
